# Caught Between a Well‐Intentioned State and a Hostile Federal System: Local Implementation of Inclusive Immigrant Policies

**DOI:** 10.1111/1468-0009.12671

**Published:** 2023-09-14

**Authors:** MARIA‐ELENA DE TRINIDAD YOUNG, SHARON TAFOLLA, FABIOLA M. PEREZ‐LUA

**Affiliations:** ^1^ School of Social Sciences, Humanities and Arts University of California Merced

**Keywords:** immigrant policy, state policy, local policy, immigrant health, safety net

## Abstract

**Policy Points**

- Inclusive state immigrant policies that expand rights and resources for immigrants may improve population health, but little is known about their local‐level implementation.
- Local actors that have anti‐immigrant attitudes can hinder the implementation of state policies, whereas the persistent influence of anti‐immigrant federal policies reinforces barriers to accessing health and other resources granted by state policies.
- Local actors that serve immigrants and support state policy implementation lack the resources to counter anti‐immigrant climates and federal policy threats.

**Context:**

In the United States, inclusive state‐level policies can advance immigrant health and health care access by extending noncitizens’ access to public benefits, workplace rights, and protections from immigration enforcement. Although state policies carry promise as structural population health interventions, there has been little examination of their implementation at the local level. Local jurisdictions play multiple roles in state policy implementation and possess distinct immigration climates. Examining the local implementation of state immigrant policy can address challenges and opportunities to ensure the health benefits of inclusive policies are realized equitably across states’ regions.

**Methods:**

To examine the local implementation of state immigrant policies, we selected a purposive sample of California counties with large immigrant populations and distinct social and political dynamics and conducted and analyzed in‐depth interviews with 20 community‐based organizations that provided health, safety net, and other services.

**Findings:**

We found that there were tensions between the inclusionary goals of state immigrant policies and local anti‐immigrant climates and federal policy changes. First, there were tensions between state policy goals and resistance from local law enforcement agencies and policymakers (e.g., Board of Supervisors). Second, because of the ongoing threats from federal immigration policies, there was a mismatch between the services and resources provided by state policies and local community needs. Finally, organizations that served immigrants were responsible for contributing to policy implementation but lacked resources to meet community needs while countering local resistance and federal policy threats.

**Conclusions:**

This study contributes knowledge regarding the challenges that emerge after state immigrant policies are enacted. The tensions among state immigrant policies, local immigration climates, and federal policy changes indicate that state immigrant policies are not implemented equally across state communities, resulting in challenges and limited benefits from policies for many immigrant communities.

In the united states, state‐level immigrant policies have become an important determinant of immigrant health and health care access.^1^ State policymakers have the authority and discretion to enact inclusive immigrant policies that extend noncitizens’ access to resources, such as eligibility for public benefits (e.g., Medicaid, nutrition programs) or in‐state university tuition; rights in the workplace, such as wage and hour standards for immigrant‐dominant industries (e.g., domestic work, agriculture); and protections from contact with the immigration enforcement system (e.g., prohibiting police collaboration with immigration authorities).^1^ These policies contribute to an inclusive “context of reception”^2^ for immigrant communities and hold promise as a structural intervention to promote population health.^3^ However, state‐level immigrant policies do not exist in isolation; they are enacted and then implemented in the context of both federal and local immigrant policies, institutions, and climates.^4^, ^5^ Little is known about the processes and practices that unfold as inclusive state immigrant policies are implemented in states’ local communities, which are embedded in the multilevel structure of immigrant policy.

In this qualitative study, we sought to understand how inclusive state‐level immigrant policies that expand rights and protections for immigrants were implemented and experienced in a selection of counties in California, a state that has enacted numerous inclusive policies in the last two decades.^6^ Although policy enactment is the process that establishes rules, regulations, or mandates for a jurisdiction, policy implementation is a “top‐down” process in which institutions and officials carry out the new rules, regulations, or mandates.^7^ Implementation can take the form of offering resources or programs or engaging in regulatory actions. States enact inclusive immigrant policies to establish goals for the treatment of immigrants across the state. However, local implementation unfolds within unique local immigration climates and through local institutions and officials who can exert discretion, as well as enact their own local ordinances or programs, which may result in variation in state policy outcomes.^7^ Through in‐depth interviews with community‐based organizations (CBOs), we observed the tensions the emerged between the goals of inclusive state policies and both local and federal policies and immigration climates. Understanding the factors that influence state‐level policies “on the ground” can inform policy development and evaluation and, ultimately, ensure equitable benefits of inclusive policies for immigrants within and across US states.

## Inclusive State Immigrant Policies Can Promote the Health of Immigrant Populations

Although only federal *immigration* policy can determine who can migrate to and their citizenship/legal status in the United States, states have the ability to enact *immigrant* policies that establish noncitizens’ rights and eligibility for resources in sectors from health care to employment to criminal justice.^5^ Mounting evidence shows that the enactment of state‐level immigrant policies is associated with health and health care access of noncitizens,^8^, ^9^ as well as populations of color who can experience the spillover effects of policy.^10^ To date, health policy researchers and advocates have primarily focused on understanding how restrictive state‐level policies influence health, such as limits to noncitizens’ eligibility for public benefits or collaborative agreements between law enforcement and federal immigration authorities.^9^ For instance, evidence shows that immigrants and Black, Latino, and Asian people in states with a greater number of restrictive policies have worse outcomes on some indicators, such as health insurance, mental health, and birth outcomes.^8^

However, states can also enact inclusive policies that extend Medicaid eligibility, driver's licenses, and in‐state university tuition to state residents regardless of legal status and that limit collaboration between local law enforcement and federal immigration authorities.^11^ Inclusive state‐level immigrant policies can promote immigrant health and access to health care by contributing to a welcoming “context of reception,” which creates community conditions that advance immigrant social, economic, and political integration.^2^ In the last couple of decades, policymakers and advocates in states with some of the largest immigrant populations, such as California, New York, and Illinois, have enacted inclusive, proimmigrant policies that aim to include immigrants in state health and social safety nets and protect them from federal immigration enforcement, which is a source of chronic stress and a barrier to health care access.^3^, ^12^ A growing body of research on inclusive policies suggests that states that have enacted more, compared with fewer, inclusive immigrant policies have better population health outcomes, particularly among Latinos, including improved access to health care and birth outcomes.^13^, ^14^

## The Need to Understand How Immigrant Policies Are Locally Implemented

Although the enactment of inclusive state immigrant policies may be a promising health policy intervention, little population health research has examined what happens as these policies are implemented across states’ diverse regions and communities. After inclusive state policies are enacted in state capitals, local institutions and public service officials implement policies in the form of new programs, resources, and rights for immigrant populations.^15^ There is some evidence that inclusive state policies are associated with varied outcomes for different populations,^16^ pointing to the need to identify implementation barriers and understand how (or if) policies translate into equitable access to new programs, resources, or rights for community residents.

Within states, local communities also possess unique “contexts of reception.” These local immigration contexts, however, do not exist in isolation. Rather, they have been described by scholars as “nested contexts of reception” because the conditions of immigrant social, economic, and political integration are embedded within broader federal and state immigration contexts.^17^ Cities and counties commonly have local policies toward immigrants (e.g., ordinances) that differ from the policy goals of federal or state governments.^4^ In addition, the mandates of state policy may be at odds with the goals or priorities of restrictive federal immigration policies (e.g., enforcement), potentially creating jurisdictional or political conflicts that can play out at the local level.^7^ For example, some cities and counties have enacted Welcoming City programs or “sanctuary” ordinances that expand health programs for immigrant residents or that reduce exposure to chronic stress through protections from immigration enforcement, varying from what is authorized under federal state policy.^18^, ^19^ There is also evidence that social and political attitudes toward immigrants vary across regions in a single state, such as between urban and rural communities.^20^ As a result, state‐level policy goals may not always align with the policies and climates nested at the local level. Examination of how inclusive immigrant policies are locally implemented—from access to state‐funded programs and services to compliance with state mandates—can shed light on and help address the challenges for ensuring that policies intended to extend resources and rights are realized equitably across populations and regions and, ultimately, contribute to contexts that promote immigrant health.

## Local “Contexts of Reception” and State Policy Implementation

Despite their limited power over federal immigration and state immigrant policy, migration scholars have long argued that cities, counties, and other local jurisdictions should be considered important sites of immigrant policymaking.^4^, ^21^ The concept of “nested contexts of reception” provides a lens to examine how the implementation of state immigrant policies may be influenced by multiple levels of social, economic, and political factors.^17^ As we discuss here, the implementation—and potential health benefits—of inclusive state‐level immigrant policies may be influenced by local‐level policymaking, the role of local institutions, and local immigration climates. These influences highlight how localities’ policy priorities, decision making, and attitudes may intersect to shape state policy implementation. Existing research points to the challenges that may arise when the “nested contexts of reception” do not align with the state‐level “context of reception.”

First, localities may influence state‐level policies through local‐level policy enactment. Few local policies are formally immigrant policies, primarily because of localities’ limited jurisdiction over immigration matters. Local policymaking directly related to the rights of immigrant residents, however, can include city ordinances about where day laborers can seek work, county‐funded programs to provide health care services to undocumented immigrants ineligible for federal‐ or state‐funded insurance, or school‐district “safe zone” policies that protect children and families from enforcement on school grounds.^22^, ^23^, ^24^ In addition, localities are also responsible for a wide range of policies that advance the budgetary, health and social services, and civic engagement priorities of local policymakers. These are not formally immigrant policy but can influence the extent to which the local context of reception for immigrant residents is inclusionary or exclusionary.

Second, localities may influence state‐level policies through their participation in state policy implementation. Many state‐level policies are dependent on local agencies for implementation.^4^ Some local agencies, such as law enforcement, are tasked with complying with inclusive policies or enforcing the mandates of restrictive policies. Federal policies can deputize local law enforcement agencies to conduct immigration arrests or establish collaborations between law enforcement and federal immigration authorities,^25^ whereas inclusive state policies that limit collaboration between law enforcement and Immigrations and Customs Enforcement (ICE) rely on local law enforcement agencies to comply with mandates, such as enacting changes in organizational practices, ending communication with ICE, and ceasing the transfer of individuals in county jails to ICE.

This is also the case with inclusive immigrant policies related to health, social welfare, and other public benefits. Local agencies—such as community health centers, health departments, or social services agencies—enroll individuals into new state programs and offer direct services funded by state policies. Such agencies are often CBOs that are part of the private, not‐for‐profit safety net sector. Scholars argue that organizational policies and practices of the private safety net sector also constitute de facto local policy.^26^ For example, a study of the immigrant legalization program under the 1986 federal Immigrant Reform and Control Act found that local immigrant CBOs played a key role in assisting immigrants with submitting applications for legalization.^27^ Another study found that, in response to a heightened anti‐immigrant climate, community health centers adjusted their policies and practices to protect immigrant patients from potential disclosure to ICE.^28^

There has been little research on local implementation of state policy because most research on immigrant policy and localities has focused on implementation of federal policy or factors that determine the creation of county or city ordinances.^15^, ^29^, ^30^ This literature, however, provides insights into the factors that may shape how state immigrant policies are received, implemented, and experienced locally. Local demographic, political, and economic factors, such as the size of the communities’ foreign‐born population, changes in the Latino population, population educational attainment, and median income, are associated with the enactment of local ordinances, budgetary priorities, and agency practices.^15^ For example, the characteristics and internal policies of local law enforcement agencies may influence their collaboration with federal immigration enforcement.^18^ One study found that law enforcement agencies with larger percentages of Latino and Black personnel had a lower number of submissions to ICE for background checks and fewer removals^29^; another found that agencies that had a no‐racial‐profiling policy also had lower removal rates.^30^

Finally, local immigration climates may play a role in state policy implementation. Immigration climates reflect political priorities and ideologies, as well as social attitudes related to migration and immigrants.^15^, ^18^, ^19^ There is considerable variation across US counties and cities in attitudes toward immigrants, as well as social and political values regarding national identity and belonging.^31^, ^32^ Evidence suggests that local attitudes and priorities around immigration do diverge from and, in some cases, actively oppose state policies. For example, in one study, localities that were Republican‐leaning had higher levels of deportations under the federal Secure Communities enforcement program.^18^ There is also evidence that residents of rural, compared with urban, communities in the United States have more anti‐immigrant attitudes.^15^, ^20^ In general, evidence suggests that community composition—from racial/ethnic diversity to economic inequality—is associated with support for policy responses to local immigrant residents.^19^, ^23^, ^33^

Overall, this research indicates that despite being “nested” in and influenced by federal and state policies, local factors influence policymakers’ priorities and actions. The variation in policies and immigration climates across communities may result in tensions between localities and the state. Such tensions have been examined in states with restrictive, enforcement‐focused immigrant policies such as Texas, where localities, usually urban areas, have enacted local “sanctuary” policies to limit law enforcement collaboration with ICE. State policymakers reacted to these local‐level policies by outlawing “sanctuary” jurisdictions and mandating law enforcement agency cooperation with ICE.^34^ However, there has not been a similar examination of the possible tensions between inclusive state‐level policies and policymaking and climates in local communities.

## Study Objectives and Context

To examine how inclusive state immigrant policies may be experienced and implemented in local communities, we conducted in‐depth interviews with CBOs in a selection of California counties. California provided an ideal “context of reception” in which to observe the implementation of inclusive state immigrant policies in local communities that likely had divergent policy priorities and climates from those of the state. California has been a leader in enacting inclusive policies, often as a direct response to restrictive federal policies, aimed at improving immigrant access to health care and other social services and protecting communities from encounters with federal law enforcement.^1^ Yet, there is evidence of local‐level variation in how immigrant communities have been treated across the state.^35^, ^36^ By purposively selecting counties that were rural and, therefore, likely underserved and politically conservative within a largely proimmigrant state, we sought to observe unique “nested contexts of reception.” We conducted interviews with CBOs that provided services and conducted civic engagement and policy advocacy under the state's policies. CBOs play a key role in the health and social safety net, providing and connecting immigrants to the services and resources authorized by inclusive immigrant policies. They also have firsthand experience observing and, in some cases, monitoring local‐level policy compliance, offering insights into the local‐level implementation of inclusive state‐level policies.

## Methods

We conducted in‐depth semistructured interviews with 20 respondents from CBOs that provided health, safety net, and other services in four counties in California from October 2020 to January 2021. The research received approval from the UC Merced Institutional Review Board.

### Selection and Recruitment of Sample

First, we purposively sampled a selection of California counties. Based on evidence of distinct political patterns between urban and rural regions,^15^, ^20^ the study team's knowledge of state regions, and input from community collaborators, we identified counties outside of the state's major urban metropolises with distinct social and political climates from the state (e.g., rural communities, voting patterns) and that were underserved and under resourced. We then identified four counties that also had large Latino immigrant populations (based on the American Community Survey). Two regions in California were identified as being the most representative of these characteristics: The San Joaquin Valley (SJV) and Imperial Valley (IV). We selected counties in the SJV (Merced, Fresno, Tulare) and IV (Imperial) from which to sample respondents. The SJV and IV have distinct geographic, social, economic, and political characteristics that shape their contexts of reception. The SJV stretches 400 miles through the heart of California, and the IV lies in the southern region of the state, bordering Mexico. Although both contain urban cores (e.g., Fresno City), most communities are rural, and residents are underserved because they have limited access to health and social safety net services compared with residents of the state's urban regions.^37^ Both have large Latino immigrant populations, with approximately 600,000 Latino immigrants in the SJV^38^ and about 400,000 in the IV.^39^ Because of its location along the southern border, US‐ and foreign‐born Latinos in the IV circulate frequently between Mexico and the United States. The economy in both regions is largely driven by agriculture,^40^ which primarily employs undocumented Mexican immigrants.^41^ The SJV and IV have similar political leanings: In 2020, both regions had a higher percentage of Republican voters compared with other regions of the state.^42^

We then identified CBOs that worked in these counties through our professional networks and internet searches. We first identified types of CBOs that should be included to obtain a diverse sample. This included health, social services, educational, and labor and/or training organizations. We generated a list of CBOs that we knew worked in these areas. The authors lived and worked in Merced County and were active in regional health and immigration collaborations that also included partners from Fresno and Tulare Counties, providing insights into which CBOs to include. Then, for each county, we conducted systematic internet searches for each of the above type of CBOs (if we had not already identified one). This included scanning the websites of selected CBOs to identify their partners and use of search terms. CBOs were eligible to participate if they provided health, safety net, or other services in the study counties. We did not seek CBOs that worked exclusively with immigrant populations; however, we anticipated that most worked in some capacity with immigrant communities because of local demographic compositions. We invited 39 organizations to participate in the study via email and invited them to select a staff member to be interviewed. Ultimately, 20 organizations agreed to participate in the study.

### Interview Procedures

We created an interview guide organized around the two major areas of inclusive state policy: the safety net and immigration enforcement policy. Questions were organized to start with topics related to each organization's service focus. For example, we asked nutrition assistance organizations about enrolling immigrant clients in and providing food assistance programs, employment/labor organizations about activities related to workers’ rights, and health care organizations about enrolling immigrant clients in public benefit programs and navigating eligibility requirements. Through open‐ended questions, we sought descriptions of their experiences implementing policies or programs, providing services, or conducting advocacy in their communities. We asked all respondents about the state's Medicaid and drivers’ license policies and policies limiting collaboration between law enforcement and ICE.

Before the interview, a research team member met with the staff representative to describe the study and obtain consent. All interviews were conducted in English via Zoom (duration: 45–60 minutes). We used Zoom's automatic transcription feature to transcribe the interviews. One research member edited the interview transcriptions for clarity and accuracy.

### Data Analysis

After each interview, research team members wrote memos and met to discuss emerging themes. When we observed that additional interviews were not yielding new topics or themes, we determined that we had reached saturation.^43^ Drawing from memos, we developed a codebook by first generating “top‐down” codes related to policy topics. Then, we coded a selection of transcripts to refine the definition of each code. At this stage, we generated “bottom‐up” codes based on topics that emerged from the transcripts. After finalizing our codebook, one member of the research team coded all interviews and met with the principal investigator (first author) and research team on a weekly basis to discuss the processes and emerging themes. Research team members created code trees to explore the relationships among code groups that formed themes. Following a community‐engaged research approach,^44^ we reported our preliminary themes to organizations working in the sample communities through a collaborative convened by the Immigrant Legal Resource Center. Reporting findings back to the community provided an opportunity to integrate community knowledge in the analysis process and confirm the saturation of themes.^44^ Our community collaborators shared how the findings reflected their experiences in the community and identified dynamics that were not represented in the study. The team then returned to the code groups to finalize the study themes. Three themes emerged from our analysis: the power and influence of local actors who held anti‐immigrant political and social views, the mismatch between state policy goals and changes in federal immigration policy, and the lack of resources for actors who supported or served immigrants to effectively implement policies.

## Results

**Figure 1:**
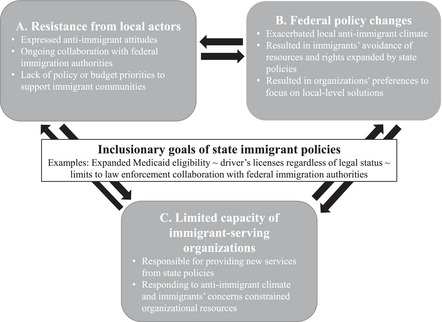
Local Implementation of Inclusive State Immigrant Policy Tensions between policy goals and (**A**) resistance from local actors, (**B**) federal policy changes, and (**C**) limited capacity of immigrant‐serving organizations.

The study respondents represented 20 CBOs that provided a range of services, including basic needs, adult education, health care or health promotion, and legal aid, and who conducted civic engagement activities in the selected counties (Table 1). All respondents reported that their CBOs provided services and resources to immigrant clients, whereas only a few had organizational missions focused exclusively on immigration‐related issues. Despite working in distinct areas, all respondents were aware of federal, state, and local immigrant policies and had observed how these influenced the local “context of reception” and their work. Most respondents’ services fell under the auspices of the state's inclusive policies, including health services covered by Medicaid or workplace safety trainings for farmworkers. Others provided services for immigrants seeking benefits or exercising rights granted by state policies, such as navigating enrollment or filing legal claims (e.g., workers’ compensation). A few others monitored policy implementation and engaged in advocacy through civic engagement of residents or policy advocacy. They reported working in largely hostile immigration climates where, despite the growing presence of numerous inclusive state policies, they contended with the actions of local leaders and policymakers opposed to inclusive immigrant policies, fluctuations in federal policy, and limited organizational resources. Three themes emerged from our analysis that describe how the “on the ground” implementation of inclusive state immigrant policies was experienced in local communities. Inclusive immigrant policy implementation was influenced by tensions between the goals of these policies and local‐level factors and changes in federal policies. Figure 1 depicts the tensions influencing local‐level state policy implementation. First, respondents described that there were tensions between the intent of the state's inclusive policies and resistance from local law enforcement agencies and policymakers (Figure 1A). Second, local communities contended with the ever‐present threat of federal policy changes (Figure 1B). As a result, tensions also emerged from the mismatch between the intent of inclusive state policies and the needs of local immigrant community members to feel safe from hostile federal immigration laws as they sought new services and resources under state policy. Finally, respondents reported that they and other organizations that provided services to immigrant residents were expected to contribute to inclusive policy implementation; however, they frequently lacked the resources to meet immediate community needs while also countering resistance to policy implementation from local policymakers and federal policy threats (Figure 1C).

**Table 1:** Study Sample Characteristics*

Organization Characteristic	*n*
Counties served	
Merced	8
Tulare	5
Fresno	2
Imperial	1
Multiple	4
Organization services/activities	
Basic needs	6
Health care	2
Health promotion	3
Adult education	2
Legal aid	2
Civic engagement	5

^*^Basic needs include food assistance, nutrition support, poverty assistance, and family resources. Adult education includes job training. Civic engagement includes, but is not limited to, housing, environment, immigrant, reproductive, and human rights.

The themes describe how state policy goals were implemented in the “nested” contexts of local political and social climates, the ongoing influence—and threat—of federal immigration policies, and limited investment in proimmigrant actors to implement state policy at the local level. Despite the aim of inclusive state immigrant policies to increase resources and rights, policies were perceived, as one respondent described it, as “a false promise” of inclusion and protection for immigrant communities. Ultimately, local factors and the intersecting tensions among local, state, and federal policies created barriers to immigrant communities benefiting from inclusive state immigrant policies, highlighting ongoing gaps in state policies.

### Tensions Between State Policy Goals and Resistance From Local Actors

Respondents reported that many local law enforcement leaders and policymakers—which we refer to as local actors—held anti‐immigrant political and social views. Respondents involved in civic engagement had documented cases in which local law enforcement agencies continued to engage in some forms of collaboration with federal immigration authorities, practices also documented by recent investigations of sheriff departments’ noncompliance with state policies.^35^ Other respondents had observed that local policymakers did not prioritize policies or funding to address immigrant community needs. In the context of these experiences, respondents expressed that the inclusive state policies had little influence on local attitudes toward immigration enforcement, surveillance, and local policy priorities—resulting in no noticeable changes to community immigration climates.

Respondents shared that many county sheriffs publicly expressed anti‐immigrant attitudes and continued to engage in enforcement practices despite the mandates of state policies, limiting the benefits of these policies for immigrants in their communities. Policies such as State Senate Bill 54, the VALUES Act, had established limits on ways that local law enforcement agencies could communicate with ICE or conduct transfers of individuals in county jails to ICE custody. Sheriff and police departments were responsible for implementing policies to limit their own collaboration with immigration authorities. Respondents had observed local law enforcement agencies take openly political stances against state policies. For example, Frances, from a health promotion organization, shared that their sheriff had explicitly opposed state policies limiting local collaboration with ICE:

The sheriff department here, Sheriff Youngblood, made very explicit comments that he was not going to oblige with the sanctuary state laws and was going to continue to hold immigrants in detention.

Respondents perceived that a lack of accountability measures in the state policies resulted in the inconsistent implementation of policies. Some respondents had been involved in local efforts to monitor policy compliance. They described how agencies had skirted the letter of the law by finding ways to continue communicating with ICE. Some also described how sheriffs’ departments avoided sharing the number of transfers from county jails to ICE in mandatory public forums—a requirement of state policy.

Furthermore, many respondents shared that their communities continued to observe a strong presence of ICE, a federal agency, and high rates of arrests and deportations. Several respondents expressed both frustration and disillusionment at the lack of significant change in local anti‐immigrant practices in their communities. Ana, from a civic engagement organization, shared the following:

People felt very disenchanted. Like their sheriffs are lying about how often they transfer people [to ICE]. They are saying it's zero when we know it's more. I think that's really frustrating.

Samantha, who was also from a civic engagement organization, noted that the policies set up a ‘false promise’ of protection for immigrants. Although the law granted individual immigrants with rights and protections if they were to be in local law enforcement custody, it could not stop ICE, a federal agency, from operating in communities. Samantha said the following:

There was some time where that [law] felt very reassuring to the community. But we've seen people continue to be arrested and detained and deported.

Local elected leaders in the study communities, such as the Board of Supervisors, city council, or the school board, were not as openly resistant to state policies as law enforcement agencies. Yet, as respondents described, the lack of policy or budgetary priorities to support immigrant communities produced an indirect resistance to the rights and resources granted by state policy. Reflecting on their local efforts to advance proimmigrant polices, Jordan, from a legal aid organization, stated the following:

The Central Valley is very conservative. So, a lot of our fights are just against that – a conservative anti‐immigrant mentality when looking at Board of Supervisors and city councils.

In contrast, respondents saw policymakers in other counties taking action. Ana noted how policymakers in other regions, particularly large urban areas such as Los Angeles, had made active efforts to oppose immigration enforcement. Ana said the following:

Some counties, like L.A. County, have a total prohibition on transfers from their department to ICE. We've tried to push [local] counties to do the same.

Most respondents were left feeling that both local and state policymakers overlooked the immigrant community. There were some exceptions in cases in which respondents observed that local elected officials were informed, or willing to learn, about immigrant‐related issues and policy solutions. In such cases, respondents’ organizations had been able to advance proimmigrant policies locally to improve protection for immigrants under state policies. For example, Jordan shared that the city of Fresno had recently experienced changes in city council leadership. New councilmembers willing to support proimmigrant policies had created a public fund to pay immigrants’ legal defense fees. This reflected how additional local‐level efforts were needed to bolster state policies.

Overall, local law enforcement agencies’ resistance to implementing state policies, the lack of policy compliance oversight, and the limited support of other local policymakers for advancing proimmigrant policies posed challenges to the local implementation of inclusive state policies. Gregorio, whose agency conducted adult education for farmworkers, captured the reality of living in a local community where the full potential of inclusive state policies could not be realized because of these challenges:

When you're an immigrant and you're residing in a sanctuary state [the policy] probably allows you to sleep a little better at night. But it doesn't change your daily life. You're still going to get pulled over if somebody wants you to be pulled over. You're still going to be frowned upon when you walk into the grocery store with dirty clothes from working in the fields. It's more for peace of mind than anything for the migrant worker.

### Tensions Between State Policy Goals and Federal Policy Changes

Respondents reported that inclusive state policies did not mitigate the anti‐immigrant climate created by federal policy; rather, the ongoing threat of changes in federal policy produced additional challenges to realizing benefits of state policies. The impact of federal policy changes had particularly detrimental consequences for the state's safety net policies that had aimed to expand immigrants’ eligibility and access to resources, such as nutrition assistance, Medicaid, and driver's licenses. Federal anti‐immigrant policies created barriers for respondents to implement these programs and services. As a result, many respondent organizations had to adapt local solutions that addressed gaps in state policy.

The proposed and then enacted changes to the federal public charge rule resulted in community members avoiding state services, even when state‐funded health and nutrition programs were not subject to the rule. For instance, Maribel, from a health promotion organization, observed a drop in Medicaid enrollment in her community following the announcement of the new public charge rule. They explained:

People were scared of public charge for a while…They stopped using Medi‐Cal, they stopped collecting CalFresh, because they were scared of possibly not becoming a citizen. They didn't understand what public charge was and that you can't be charged for public charge if you're a resident or have some type of status and if you don't have status.

Stacy, from a basic needs organization, observed a “mass exodus from CalFresh” (California's Supplemental Nutrition Assistant Program) after the announcement of the new rule. For their organization, the mass exodus “illustrated how many families here are dealing with immigration issues.” Samantha also expressed concern about immigrants in their community not using the critical services for which they were eligible. They shared the following:

I haven't seen people reporting being turned away or denied services. My major concern is the opposite: people not seeking the services and trying to access services.

In addition, federal policy threats further compounded the anti‐immigrant climate produced by the practices, described above, of local law enforcement. Concerns about immigration enforcement from either federal authorities or local law enforcement further contributed to community members’ avoidance of services. Martha, whose organization provided basic needs, described how rumors of immigration arrests and disclosure of private data at social service offices circulated in their community:

We found a lot of rumors in the immigrant community. ‘I heard that [ICE] just went to welfare and picked up 55 people.’ There was no ICE there. There were a lot of rampant rumors going around for a while…[such as] that [organizations] knew who was undocumented based on how they were put into [organizations’] database.

Federal policy changes also produced challenges for implementation of state programs intended to increase immigrant residents’ rights and protections. California enacted AB60 to increase access to driver's licenses by eliminating legal status eligibility requirements. However, the federal REAL ID Act undermined the state policy, producing concerns about seeking driver's licenses among immigrant residents. Community members questioned whether it was worth carrying a driver's license that could identify them as undocumented. Frances explained:

Now we've seen increasing fear around the REAL California ID. The sheriff department and police departments have already said that they are going to continue to be non‐compliant with the sanctuary state laws and policies. Our LGBTQ BIPOC communities are already targeted for being visibly queer or trans and people of color and Black. [For them] it's better to just not have an ID at this point.

In addition, within the hostile federal policy climate, many respondents observed that bureaucratic burdens at state agencies, such as the Department of Motor Vehicles (DMV), resulted in hesitance to access resources. Despite making driver's licenses available, numerous respondents expressed concern that the state's policy did not address language access or literacy limitations that were specific to immigrant residents. Furthermore, others described how complex bureaucratic processes compounded concerns about the value of obtaining a license. For example, Samantha reported how clients struggled to meet administrative requirements to obtain a driver's license. They explained:

There have been some issues at local DMV offices with people getting their AB 60 licenses, like what documents they are required to have. Or when people newly receive a benefit, and then go back and try to transfer from an AB 60 to a regular standard license, sometimes we have seen issues at the local DMV level.

Respondents observed that, just as state policy was extending eligibility and access to services, community members were avoiding seeking or utilizing services because of federal immigration policy concerns. The state's inclusive immigrant policies could not mitigate the confusion and misinformation created by the national‐level anti‐immigrant climate and federal policy changes, nor, as we discuss more below, did CBOs have the resources to meet the need for outreach, education, and navigation support.

Many respondents reported that, like their clients who avoided state‐funded services, their organizations also disengaged with state policies because of the barriers created by federal policy. Instead, they chose to focus on organizational policies and local‐level policies that could be tailored to community needs. Respondents modified their organizational policies to be more inclusive of immigrants in areas where state policies failed to address federal immigrant exclusions. The strategies employed by some CBOs were resource intensive yet could address immigrant community member's concerns. For example, organizations accepted various forms of identification, such as school IDs, to ensure that identification was not a barrier to their services. Meredith, from a health care organization, said, “We will accept a lot of different IDs at [our organization]. We don't require a REAL ID or anything like that. So, we're pretty easy.” Local organizations also became involved in setting policy priorities within local communities. Their experience of state policymaking was that their local political climate and needs were often not considered when the state set policy priorities. Respondents noted that they preferred to be politically involved at the local level rather than the state level because it was a better opportunity to achieve local solutions. Cheryl, from a basic needs organization, explained:

We try to keep it local. We try to keep our relationships open with the board of supervisors with city council members. We are really looking at local solutions. Because, like I said, there's such a difference between the [SJV's] culture and the rest of the state's culture. Most of the time what happens in Sacramento doesn't reach us or makes it worse here for us.

Changes to federal policies, such as the expanded public charge rule and REAL ID, created uncertainty among immigrants in local communities and resulted in avoidance of resources available under state policies and in their communities. Although inclusive state policies expanded immigrants’ rights, protections, and access to services in their communities, state policies were unable to address the hostile conditions created by federal policies, highlighting additional tensions among local, state, and federal levels.

### Tensions Between State Policy Goals and Limited Capacity of Local Actors That Serve Immigrants

Respondents and their organizations, as well as others that provided services to the immigrant community, took the role of local actors who could contribute to the implementation of inclusive immigrant policy. However, within the anti‐immigrant “context of reception” created by some local actors and federal policies, they faced significant limitations that hindered their ability to improve immigrants’ access to new services, resources, and rights.

Organizations worked to advance inclusive state policy implementation by extending their eligibility criteria to services, connecting entire mixed‐status families to resources through eligible family members, and referring clients to partner organizations. However, organizations’ capacity to serve immigrants was restricted by their limited resources. Organizations found themselves dedicating significant staff time—often beyond their funded scope of work—to simply building trust in the community and ensuring clients understood their rights and eligibility.

Outreach typically took the form of educational workshops on public charge or how to access different resources. It also included distributing hand‐sized “red cards” that explained an individual's rights if they encountered ICE, hosting trainings on immigrant rights, and connecting clients to legal aid or financial resources. Maribel shared the following:

We've been really vocal about people's rights, presentations and sharing the little red cards that have your information in case something happens. We don't want our community to be scared.

As they attempted to build trust and inform the immigrant community, respondents themselves had limited time and resources to keep up with the frequent changes in policy that could contribute to fear, misinformation, and confusion. Samantha shared their concerns after providing an informational workshop to immigrant youth:

I worked with the Mexican consulate in Fresno. We did a Facebook live with them on DACA [Deferred Action for Childhood Arrivals]. The next day was the court order [on DACA] and everything I had said no longer accurate. It's really hard then to go back to the community and say, ‘oh, yeah, it was wrong what I said. Not because it was wrong in that moment, but because things are evolving so rapidly.’

Similarly, local immigration attorneys faced challenges to providing services and information to immigrant clients. Some respondents observed that frequent policy changes led attorneys, some private and others at nonprofit legal aid agencies, to inadvertently spread misinformation about policies. Melissa, from a health care organization, described how misinformation about the public charge rule influenced one of their clients’ decision to refuse benefits:

Even after showing [the client] the information [about Medi‐Cal eligibility], even after printing it up, he still said, ‘No. I need to go to a place where I can at least have a payment plan because my attorney said we couldn't get any government help at all.’

Given the challenges of disseminating information about state and federal policies, many community members looked to the Spanish‐language media for information about immigration policies. Respondents, however, expressed concerns about the accuracy of information shared. Conchita, from a civic engagement organization, shared the following:

People know about the big [policies] through Univision, Telemundo, media, I think. The work that we do as a CBO is giving immigrants a deeper understanding. But we hear there are gaps: how do we put something together to cover as many people as possible?

Another respondent shared that media often inadvertently amplified concerns that federal policy posed a threat in local communities. Exposure to news stories about major immigration enforcement events outside their communities created the perception that enforcement activities were taking place close by. For example, coverage of ICE raids in other parts of the country had had a negative effect on the local community. Stacy shared the following:

The ICE raids started happening in other places in the country. That was the top of the news here for a long time. It just instilled that fear in those immigrant families that have issues.

Despite efforts by local organizations to support and implement state sanctuary policies, organizations’ time and efforts were consumed by the need to address misinformation and fear created by an anti‐immigrant federal climate. Other local actors, such as private attorneys and local Spanish‐language media, could, at times, exacerbate misinformation. Organizations’ capacity was limited by a lack of resources to combat the anti‐immigrant climate and implement state policies. As one community member commented during our report‐back sessions, “We are caught between a well‐intentioned state and a hostile federal system.”

## Discussion

In this study, we conducted in‐depth interviews with representatives from CBOs that served immigrant communities in four California counties to examine how the state's inclusive immigrant policies were experienced “on the ground.” We found that, despite the state's inclusionary policy goals to expand resources and rights for immigrant residents, policy implementation intersected with local “nested contexts of reception” that were shaped by both local actors and the threat of federal policy change. Local law enforcement agencies and policymakers could hinder the implementation of state policies that were intended to protect immigrants from enforcement, whereas the persistent influence from federal policies reinforced barriers to accessing the health and other safety net resources granted by state policies. Ultimately, the organizations that provided the services and resources granted by state policies lacked the capacity to counter the anti‐immigrant climate or the federal policy threats.

This study contributes to the growing research on immigration and immigrant policies as social determinants of health by looking at the challenges that can emerge after inclusive state policies are enacted. Growing evidence indicates that inclusive immigrant policies across US states have the potential for improving immigrant health and health care access.^8^, ^10^ Currently, 17 states beyond California provide Medicaid coverage to pregnant undocumented women, 21 grant in‐state tuition to undocumented students, and 15 have extended labor protections to workers in immigrant‐dominated labor sectors.^1^ Inclusive immigrant policy has been identified as an important strategy to advance immigrant health.^45^ Yet, despite the proliferation of inclusive policies and research examining the relationships between state‐level policy and health outcomes,^3^, ^8^, ^9^, ^10^, ^46^ there has been less research that examines the processes by which state policy is implemented. Understanding what happens during policy implementation is critical because policy implementation is often a “top‐down” process in which federal and state policies mandate or authorize local institutions with managing programs and practices that shape immigrant integration.^7^ By focusing on a state that had enacted numerous inclusive policies but had significant variation in local policy and immigration climates, our findings show how the multilevel nature of immigrant policy results in challenges for realizing the goals of state policy. Our findings also suggest that state‐level policies are not implemented equally across local communities. Indeed, one respondent described California's policies as a “false promise” of protection for immigrants, pointing to the unrealized potential of some inclusive immigrant policies to provide equitable benefits across the state's counties. In the following sections, we discuss the key tensions that emerged between the inclusionary goals of state‐level immigrant policies and the social and policy realities in local communities. We also highlight possible lessons for the development and implementation of future inclusive state immigrant policies.

### The Role of Local Actors

First, our findings point to the importance of understanding the role of local actors in the development and implementation of state immigrant policies. In our sample communities, local policymakers, agencies, and CBOs all played a role in influencing how state policy was implemented and experienced “on the ground.” Local actors could hinder or help the implementation of state policy. Actors who supported or served immigrant communities could ensure that new programs, such as the expansion of Medicaid to undocumented California residents, or rights, such as worker protections, reached immigrant residents to, ultimately, improve their health. However, other actors who held anti‐immigrant views or who simply did not prioritize the needs of immigrant communicates could interfere with policy implementation.

Our findings regarding the role of local actors align with research on what is referred to as “street‐level bureaucrats,” which recognizes that public policies are, ultimately, implemented by agency or institution personnel who have direct contact with service recipients (e.g., community members, clients, patients).^26^ Because policy implementation is dependent on the compliance and accountability of street‐level bureaucrats, the actions of individual actors becomes de facto policymaking.^47^ Indeed, we observed that the reality of state immigrant policies “on the ground” was shaped by how agencies, such as the sheriff's department, reacted to policies and, in turn, community members’ perceptions regarding the safety of accessing services.

Previous research on street‐level bureaucrats and immigrant communities has primarily focused on how agency personnel can create pathways of inclusion for immigrants within broader anti‐immigrant contexts. For example, previous studies in new immigrant destinations and in California showed how local police departments created community policing and education programs to incorporate immigrant residents into the community through supportive and positive interactions with local agency personnel.^48^, ^49^ In contrast, our findings highlight the dynamics by which local agencies and their personnel contribute to immigrant exclusion by hindering the implementation of inclusive policies. Local actors, such as law enforcement, have considerable power to directly thwart policies through noncompliance. Even when local actors, such as a city council or Board of Supervisors, were not overtly anti‐immigrant, a lack of investment in immigrant communities reinforced an overall anti‐immigrant environment. Finally, although immigrant‐serving CBOs had a clear role to play in delivering health and other services under state policies, their discretion was often impeded by limited resources. Indeed, in our study communities, the local actors who served immigrant communities had the least resources yet were the actors most committed to ensuring that state policies translated to improved rights, protections, and climate for immigrant residents.

Our findings also suggest that, despite enacting inclusionary goals, inclusive state immigrant policies do not address the inequitable power and resources of different local actors. The tensions that we observed between state policy goals and resistance from some local actors and the limited capacity of other local actors ultimately points to an underlying inequity between policy mechanisms that can promote immigrant inclusion and those that authorize immigrant exclusion. As we observed, immigrant policy implementation involved both service delivery by various service organizations and limits to law enforcement practices. The local actors responsible for each generally held inequitable levels of policy influence and resources.^26^, ^47^ Despite being responsible for policy implementation, local law enforcement agencies, and implicitly local elected officials, used discretion to signal limited support and not comply with state policies. In contrast, the local actors responsible for providing resources and services under state law had limited resources to both contend with service provision and counter the impact of anti‐immigrant actors.

Within these dynamics, we see the tensions between localities’ roles in policy regulation and in policy service provision.^26^ This suggests that barriers to fully realizing the benefits of inclusive state policies may emerge when localities can influence both immigrant exclusion and inclusion. For example, in ethnographic observation of the Nashville Police Department, which attempted to both conduct policing and engage in community programs for immigrants, Armenta observed this tension and contradiction.^25^ She found that the department devoted resources to a prized community policing program while patrol offers continued to routinely arrest Latino residents for minor infractions, such as failing to carry identification.^25^ She suggests that the tensions between inclusion and social control cannot be reconciled.

Given the roles of distinct local actors, state‐level policymakers should consider the policies and climates in local “contexts of reception” that may make it challenging to implement policies intended to increase immigrants’ access to the safety net and expand their rights. This includes developing systems of accountability to ensure that policies can be implemented despite local resistance, particularly when implementation requires compliance by agencies that are mandated to change or curtail activities. Policymakers at state as well as local levels can provide dedicated funding to local organizations so that they can effectively support policy implementation; this could include funding for organizations to conduct local outreach, education, and media communications about rights and services granted under the state's immigrant policies. A small body of literature highlights the importance of having a robust local infrastructure of immigrant‐serving CBOs. One study looked at the relationship between the presence of nonprofit legal aid organizations and the level of deportation in counties. Authors found that regions with a greater number of these critical organizations had lower removal numbers.^50^ Ultimately, the inequitable power, resources, and influence of local actors may be a significant contributor to varied state policy implementation.

### The Ongoing Influence of Federal Policies

We also found that the federal immigration climate continues to be an influence on local “contexts of reception,” regardless of state and local policies. The continued influence of federal policy in local communities further reinforces the importance of understanding and addressing the intersection of the multilevel and nested nature of immigrant policy. On the one hand, this is not an entirely surprising finding. It is consistent with numerous studies that show that national‐level policy, as well as media coverage and political rhetoric around federal policies, have an influence on social climates and health. For example, studies after the launch of the Trump presidential campaign found changes in health during periods of heightened anti‐immigrant sentiment.^51^, ^52^ On the other hand, this finding brings attention to the limits of state policy due to being embedded in a federal context. Many state immigrant policies are reactions to federal policy,^4^ such as efforts to expand eligibility for federally funded programs or to limit the presence of federal immigration authorities in the state. It is at the local level, however, where the tensions between federal exclusion and state inclusion play out.^19^, ^30^, ^50^, ^53^ In our study communities, we found that federal policy bolstered the anti‐immigrant climates, which were intentionally further amplified by local actors as well as unintentionally by local media. As state policymakers consider how to react to federal policies, recognition of and investment in local “contexts of reception” to counter the ongoing influence of federal policies and national‐level immigration climates is critical.

### Limitations and Future Directions

This study provides a foundation for future research on the role of immigrant policy implementation in influencing the health of immigrant populations. It has some limitations that can be addressed in future studies. First, although California provided a context in which we could observe tensions between the goals of inclusive policies and anti‐immigrant local climates, the setting may not be generalizable to other US states. California has a longer‐residing immigrant population than many states, and the local communities examined were largely agricultural. Future studies can examine other states that also have numerous inclusionary state policies, such as Illinois, New York, and Washington, but distinct immigration patterns to understand the policy dynamic across state and local contexts. Second, our respondents came from CBOs with a high level of awareness of immigrant policy issues and that, despite the barriers they described, largely served immigrants who had accessed services or were engaged in their communities. Future studies could also include institutions or organizations who provide services or resources to immigrant populations that may be hesitant to use state‐supported resources, such as private clinics or attorneys, and could include individual immigrants who have avoided or opted out of state‐funded services. Finally, although our sample provided a range of perspectives from different organizations and counties, we did not conduct comparative analyses within the sample. Comparative analyses among communities could shed light on the community characteristics, such as rurality, demographic composition, or political dynamics, that influence local actors and the overall immigration climate. Similarly, comparisons among different types of organizations, such as health centers (which have a mandate to provide publicly funded services) and local policy advocacy organizations (which are involved in policymaking), may further shed light on the dynamics observed in this study.

Overall, our findings provide a critical starting point for additional research on how local‐level policies, actors, immigration climates, and other factors matter in immigrant policy implementation.^4^ Future research can assess the patterns of “incomplete” or varied policy implementation within states. Future population studies can examine the relationships among local factors, such as demographic composition or characteristics of local leadership (e.g., sheriff and Board of Supervisors), develop measures of policy implementation, and assess the extent of implementation of state‐level policies.

## Conclusion

Inclusive state immigrant policies hold promise to promote population health, but both knowledge and policy gaps remain as barriers to ensuring that immigrants across state regions benefit. Understanding and addressing the local factors that may hinder the local implementation of inclusive state policies, such as the role of local actors, the barriers created by federal policies, and the lack of investment in agencies contributing to policy implementation, may ensure equitable implementation of inclusive immigrant policies.

*Funding/Support*: This study was supported by the California Initiative for Health Equity and Action.

*Conflict of Interest Disclosures*: The authors have no conflicts of interest to disclose.

*Acknowledgments*: We thank the many organizations that supported this study and shared their time to discuss their experiences.
